# *In vivo* pink-beam imaging and fast alignment procedure for rat brain lesion microbeam radiation therapy

**DOI:** 10.1107/S0909049510006667

**Published:** 2010-03-20

**Authors:** Raphaël Serduc, Gilles Berruyer, Thierry Brochard, Michel Renier, Christian Nemoz

**Affiliations:** a European Synchrotron Radiation Facility, F38043 Grenoble, France

**Keywords:** synchrotron microbeam radiation therapy, pink-beam imaging, alignment procedure, preclinical brain radiosurgery

## Abstract

A fast 50 µm-accuracy alignment procedure has been developed for the radiosurgery of brain lesions in rats, using microbeam radiation therapy. *In vivo* imaging was performed using the pink beam (35–60 keV) produced by the ID17 wiggler at the ESRF opened at 120 mm and filtered. A graphical user interface has been developed in order to define the irradiation field size and to position the target with respect to the skull structures observed in X-ray images. The method proposed here allows tremendous time saving by skipping the swap from white beam to monochromatic beam and *vice versa*. To validate the concept, the somatosensory cortex or thalamus of GAERS rats were irradiated under several ports using this alignment procedure. The magnetic resonance images acquired after contrast agent injection showed that the irradiations were selectively performed in these two expected brain regions. Image-guided microbeam irradiations have therefore been realised for the first time ever, and, thanks to this new development, the ID17 biomedical beamline provides a major tool allowing brain radiosurgery trials on animal patients.

## Abstract 2

A fast 50 µm-accuracy alignment procedure has been developed for the radiosurgery of brain lesions in rats, using microbeam radiation therapy.

## 1. Introduction

For the last two decades, several preclinical studies have highlighted the potential role of synchrotron light for medical applications and particularly for brain radiosurgery (Bouchet *et al.*, 2010 ▶; Laissue *et al.*, 1998 ▶; Serduc, Bouchet *et al.*, 2009 ▶; Serduc, Christen *et al.*, 2008 ▶). Microbeam radiation therapy (MRT) (Slatkin *et al.*, 1992 ▶) was first dedicated to preclinical brain tumor treatments. It is based on the spatial fractionation of the incident X-ray beam into an array of quasi-parallel microbeams (a few tens of micrometres wide). This unique irradiation geometry allows high dose deposition (hundreds of Gray) in rodent brains without inducing severe tissular damages when delivered unidirectionally (Laissue *et al.*, 1998 ▶; Slatkin *et al.*, 1995 ▶; Serduc, van de Looij *et al.*, 2008 ▶; Serduc *et al.*, 2006 ▶). However, therapeutic applications require several irradiation ports that might impair normal brain structures contiguous to the radiation target. Previous works revealed that lesion targeting used in preclinical experiments must be now adapted to clinical standards (Bouchet *et al.*, 2010 ▶; Laissue *et al.*, 1998 ▶; Serduc, Brauer-Krisch *et al.*, 2009 ▶) and that image-guided irradiations would considerably increase the therapeutic index of MRT in cancer treatment. Furthermore, high-precision alignment of the irradiation fields opens new perspectives in preclinical neurosciences. For instances, high-dose MRT-induced focal necrosis of a clearly identified epileptogenic focus or subthalamic nucleus might be very informative in epilepsy or Parkinson pathologies.

The biomedical beamline (ID17) of the European Synchrotron Radiation Facility (ESRF, Grenoble, France) is devoted to the applications of synchrotron radiation to the medical domains such as X-ray imaging and radiotherapy. The know-how acquired in imaging has permitted a new image-guided method for animal positioning and alignment for brain MRT to be developed. The white beam produced by the ID17 wiggler has been used for radiographic imaging, and a specially developed computer-interfaced system has allowed precise irradiation field definition and radiation target positioning in the rat head within ∼50 µm accuracy, based on skull structures.

## 2. MRT principle review

**Figure 1:**
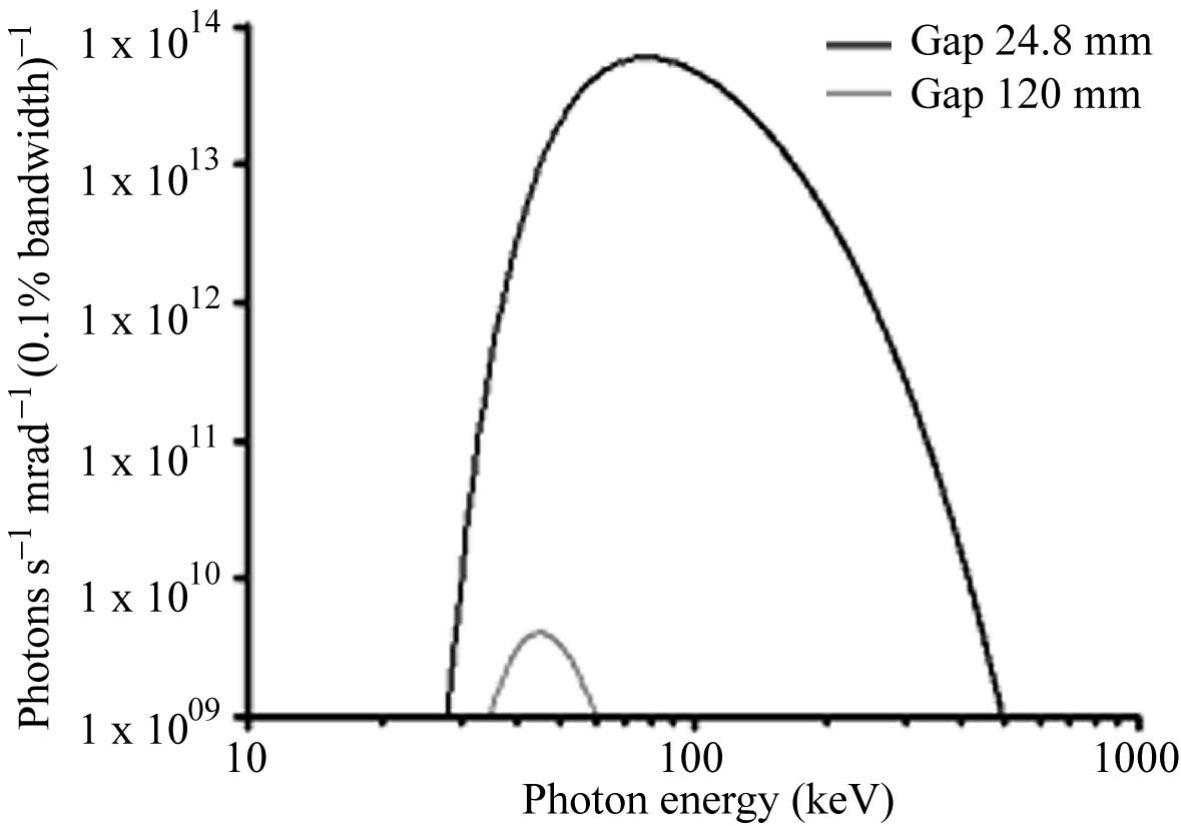
Photon beam spectra for the two considered apertures of the ID17 wiggler. Wiggler gaps of 24.8 and 120 mm (1.6 T and 0.13 T magnetic field of the 21 pole, 15 cm-period wiggler) are used for therapy and imaging, respectively. The increase in the wiggler gap aperture from 24.8 to 120 mm reduces the photon flux by a factor of 6 × 10^3^.

Microbeam irradiations of rat brain were performed at the ID17 dedicated hutch located at the front part of the beamline. For this application, the wiggler source (21-pole wiggler, 15 cm period, 1.6 T maximum field), located at 41 m from the sample, produces a white spectrum of photons which extends after filtration from 50 to 350 keV with a maximum intensity at 83 keV (Fig. 1 ▶) (Bräuer-Krisch *et al.*, 2003 ▶). Only the central cone of the synchrotron beam (0.05 mm high, 4 cm wide) was selected by calibrated slits. The quasi-laminar beam was then spatially fractionated into an array of quasi-parallel microbeams by using an adjustable multi-slit collimator (Bräuer-Krisch *et al.*, 2005 ▶, 2009 ▶; Archer, 1998 ▶) positioned about 80 cm upstream from the head of the animals. Microbeams used in MRT can vary between 25 and 100 µm in width and the distance between two microbeams is typically 100–400 µm. The dose rate in the air reaches ∼16000 Gy s^−1^. For preclinical studies, the animals are fixed on a high-precision goniometer and are vertically scanned through the microbeam array in order to restore the second dimension of the irradiation field from the original horizontal laminar beam. The goniometer vertical motor is remotely controlled and the translation speed, which determines the X-ray dose delivered to the tissues, is kept constant to within a few percent by a feedback system. The vertical field of irradiation is determined by hardware synchronization between the opening of two fast shutters and the vertical motion encoders. Adjustment of the dose is made by calibrating the goniometer speed with respect to the intensity of the electron current circulating in the storage ring. A one-port irradiation lasts ∼20 ms with a typical speed of 20 mm s^−1^.

## 3. Imaging, alignment and irradiation protocols

### 3.1. Imaging method

A two-dimensional X-ray imaging detector is installed about 2 m downstream of the animal goniometer to accurately align the samples with respect to the microbeams. The detector is a tapered optic fast-readout low-noise (FReLoN) CCD camera developed at the ESRF for a broad range of applications; its specifications were initially tailored for computed tomography in diffraction enhanced imaging mammography (Bravin *et al.*, 2003 ▶). The CCD is a 2048 × 2048 pixel chip from Atmel, USA. The size of the chip is 30 mm × 30 mm and the pixel size is around 14 µm × 14 µm. This CCD chip is coupled with a tapered optic which is a bunch of optical fibres permitting enlargement of the field of view of the detector. Finally the main spatial characteristics of this detector, which is a standard at ID17, are a field of view of 95 mm × 95 mm and a pixel size of 47 µm × 47 µm, which are fully reasonable for the alignment precision required for small-animal *in vivo* irradiation. The impinging X-rays are converted to visible light thanks to a thin gadolinium-based fluorescent screen flattened on the taper entrance. A complete study of this detector can be found by Coan *et al.* (2006 ▶).

The main positioning uncertainty of the sample with respect to the irradiation field comes from the limited spatial resolution of the detector. Indeed, the motorized positioning devices have been designed to meet the requirements of the interlaced geometry irradiations (Serduc *et al.*, 2010 ▶): the lateral parasite displacements during the vertical motion are not more than 5 µm. This precision has been confirmed by irradiating X-ray-sensitive film in the interlaced geometry mode. Other positioning uncertainty is due to motion of the animal. However, the animal is anesthetized and the contention system is sufficiently rigid to ensure a good immobility.

The detector obviously cannot endure the high levels of radiation used for MRT and the characteristics of the beam must therefore be softened. Until now, this was done by inserting a double Laue monochromator into the beam path before the target. Another option to reduce the photon flux is to open the wiggler gap until the detector signal falls within its dynamic range for a minimum exposure time. This latter solution was selected and completed by filtering the incident beam with thin metal foils. A first set of aluminium filters (*e.g.* 3 mm thickness), protected by a 1.5 mm-thick carbon filter, permits the low energy part of the spectrum to be removed. It is followed by a set of copper filters whose thickness may be adjusted from 0.5 to 6 mm. Tuning the balance of the wiggler gap aperture (120 mm, corresponding to a 0.13 T field) and the copper thickness (1.3 mm), we obtained a so-called ‘pink beam’, as shown in Fig. 1 ▶, where both spectra (calculated using the *X-ray* code developed at ESRF^1^) for therapy (gap aperture 24.8 mm) and imaging (gap aperture 120 mm) are displayed. For this latter value one can see that the remaining beam extends from 35 to 60 keV which is appropriate for small-animal imaging at conventional radiographic doses (Le Duc *et al.*, 2000 ▶).

**Figure 2:**
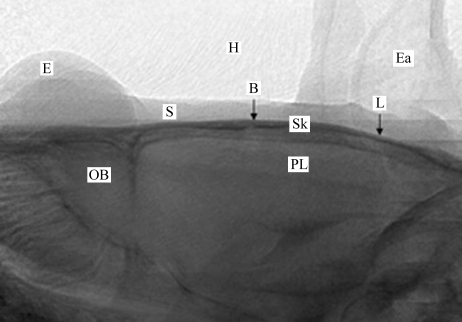
A typical *in vivo* beam-eye-view X-ray image obtained for a rat head using our imaging methods. The principal anatomical characteristics are clearly visible: eye (E), skin (S), hair (H), ears (Ea), olfactory bulb (OB) and parietal lobe (PL). Furthermore, the skull surface (Sk), the bregma (B) and lambda (L), reference points used in the rat head atlas for stereotactic surgery, are easily located (arrows).

As described in the previous section, the beam issued from the wiggler is horizontal and laminar. When all the slits are removed from the beam, its dimensions are typically 40 mm horizontal and 1 mm vertical. In order to obtain a two-dimensional image the animal is vertically scanned through the beam. An acquisition is triggered on the camera at each vertical position of the goniometer, with a 1 mm step. The acquired frames are then piled-up in order to restore the vertical dimension. A typical *in vivo* image of a rat brain is shown in Fig. 2 ▶. The vertical field of view is almost 20 mm (20 frames). The typical exposure time per frame is 0.1 s. Including the CCD readout time and the vertical motion operation time, the total acquisition time of an image is less than 20 s. As shown in Fig. 2 ▶, one can clearly distinguish in this image the eyes, the ears, the skull or the olfactory bulb, and also the bregma which is the reference point in rat brain atlas (Paxinos & Watson, 2004 ▶) and which is used for all brain radiosurgery procedures.

### 3.2. Multi-slit calibration and central pixel definition

**Figure 3:**
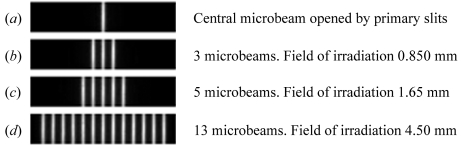
Snapshot of the detector showing the beam across the multi-slit collimator for different openings of the horizontal primary slits which delimit the irradiation field.

To precisely define the irradiation field, a multi-slit collimator (Bräuer-Krisch *et al.*, 2009 ▶) is brought into the beam. By adjusting the horizontal offset and the horizontal aperture of the primary slits located at the beginning of the beamline, one is able to define the central microbeam as shown in Fig. 3(*a*) ▶. Knowing the incident beam geometry makes it consequently easy to compute the horizontal aperture of the primary slits in order to cover the desired horizontal irradiation field (HIF) containing an integer number of microbeams as shown in Figs. 3(*b*) (three microbeams for 850 µm HIF), 3(*c*) (five microbeams for 1.65 mm HIF) and 3(*d*) ▶ (13 microbeams for 4.85 mm HIF). The maximum field corresponds to the maximum beam size, *e.g.* 100 microbeams for a 40 mm HIF. The central pixel is then defined by the centroid of the central microbeam profile. The lateral adjustment (*y* direction) of the brain zone to be irradiated will be defined around this point in the graphical user interface described in the following section.

### 3.3. Alignment method

**Figure 4:**
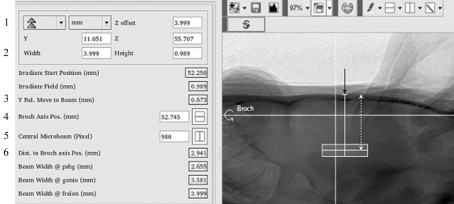
Graphical user interface used for irradiation field positioning in the rat head. The horizontal white line represents the beam and the rotation axis positions (defined by line 4). The vertical white line corresponds to the central microbeam *y* position (defined by line 5). The width and height of the irradiation field (grey on the X-ray image) are defined by line 2. For line 1, the user can also indicate an offset distance (4 mm from the skull surface in this case, white dashed arrow) between the centre of the irradiation field and a given anatomical reference (bregma, black arrow). Two motor motions in the *y* and *z* directions are required to move the target into the beam. These are automatically calculated when positioning the radiation target and are given by line 3 (*y* motion) and line 6 (*z* motion).

A dedicated graphical user interface has been developed to control both the imaging and the radiation therapy. A screen shot of the imaging part of this application is depicted in Fig. 4 ▶. The beam position height and the pixel number on the camera corresponding to the centroid of the central microbeam (previously defined) are manually reported in the X-ray image (horizontal and vertical white lines, respectively, in Fig. 4 ▶). The operator then defines the irradiation field size and the radiation target can be freely moved on the X-ray image. For any position of the radiation target the software calculates the *z* and *y* distances from the centre of the radiation target to the beam position and to the central microbeam pixel, respectively (distances given by lines 3 and 6 in Fig. 4 ▶). These distance corrections will be applied to the *z* and *y* motors in order to align the centre of the radiation target defined in the rat head with the beam height and the central microbeam of the array (symbolized by the intersection point of the two perpendicular white lines in the X-ray image in Fig. 4 ▶). No corrections are carried out on the *x* axis because it corresponds to the beam direction.

### 3.4. Imaging to therapy mode transition

Dedicated software which drives all the useful motors has been developed to swap from the imaging to the therapy mode. The FReLoN camera is protected from high radiation doses delivered during therapeutical irradiation by moving it into a lead-shielded box. In addition, the program adds a 50 µm vertical slit for beam height definition and closes the primary slits to the central microbeam position (as shown in Fig. 3*a* ▶). In the meantime, the wiggler gap is closed to its irradiation position, namely 24.8 mm at the ESRF-ID17. The control program then sets the irradiation field size in the *y* direction by modifying the number of microbeams. The symmetric opening of the primary slits (procedure described in §3.2) adds the required number of microbeams on each side of the central microbeam and defines the final radiation target size. Moving from imaging to therapy modes lasts for less than one minute.

## 4. Biomedical applications

As suggested in the *Introduction* [Sec sec1], the precise alignment procedure developed in this work opens new perspectives in preclinical neuroscience. Indeed, until now MRT was limited to brain tumor treatment but homogeneous irradiation at high doses delivered to a specific brain structure will induce macroscopic necrosis and would give important information on the role of such brain regions in neurological pathology. It has been supposed that the somatosensory cortex would initiate the spontaneous epileptic seizures in the genetic absence epilepsy rats from Strasbourg (GAERS) while the thalamus would be involved in their control (David *et al.*, 2008 ▶; Polack *et al.*, 2007 ▶). Based on stereotactic coordinates, we performed three-dimensional bilateral interlaced irradiations (Serduc *et al.*, 2010 ▶) in the somatosensory cortex and thalamus of GAERS in order to inactivate these two brain regions.^2^ The roles of the somatosensory cortex and thalamus in epileptic seizures are being studied weekly by electroencephalograms for several months and will be described elsewhere.

The irradiation field alignment was performed by using the procedure described in this study. The GAERS rat heads (*n* = 10) were imaged using pink-beam imaging and the radiation targets were then delimited on the graphical user interface. The coordinates of the centres of the irradiation fields for the cortex were ±5 mm laterally from the medio-lateral (ML) line (left and right hemisphere irradiations), −2.5 mm in the dorso-ventral (DV) below the skull surface and −0.88 mm in the anteroposterior (AP, *e.g.* from the tail to the nose of the animal) direction from the bregma (noted ‘B’ in Fig. 2 ▶). The irradiation field size was 6.15 mm × 2 mm × 2 mm. For thalamus irradiation, the coordinates were −2.6 mm ML, ±5.3 mm DV and −2.7 mm AP (irradiation field size 1.9 mm × 1.5 mm × 1.5 mm). All irradiation coordinates were chosen according to David *et al.* (2008 ▶) who showed that the S1 cortex and thalamus are activated during seizures in the GAERS rat model. The irradiations were performed across four entrance ports by rotating the animal every 45° around its antero­posterior axis (‘Broch’ in Fig. 4 ▶) hence providing four interlaced arrays of 50 µm-wide microbeams spaced by 200 µm on-centre. This operation was then repeated to irradiate the symmetrical structure target. The in-microbeam entrance dose was 200 Gy. It has been shown that the irradiation geometry used in this example induces a blood brain barrier disruption and contrast agent diffusion in the rat brain confined to the radiation target (Serduc *et al.*, 2010 ▶).

**Figure 5:**
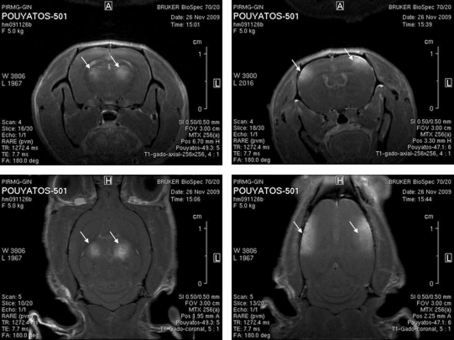
MR imaging of the targeted brain regions in GAERS. T_1_-weighted images acquired 5 min after intravenous injection. Gd-DOTA injection of the evolution of the radio-induced lesion at day 15 after irradiation. The thalamus and the somatosensory cortex were precisely targeted using our alignment procedures (arrows).

Fig. 5 ▶ shows T_1_-weighted magnetic resonance (MR) images acquired 15 days after the irradiations in the cortex and the thalamus of the GAERS rats. MR imaging experiments were performed at 7 T (Bruker Avance III system) using a quadrature volume coil. MR imaging experiments were performed at the MRI platform of the Grenoble Institut des Neuro­sciences (Grenoble, France). After anesthesia, a catheter filled with heparinized saline was inserted into the dorsal tail vein of the animal. Radiation-induced changes in brain vessel permeability were then characterized using a T_1_-weighted MR sequence (RARE sequence; TR: 950 ms, effective TE: 7.7 ms, FOV: 3 cm × 3 cm, matrix: 64 × 64, 0.5 mm-thick) acquired 5 min after an intravenous injection of Gd-DOTA (200 µmol kg^−1^). The contrast agent diffusion (hypersignal) revealed that the irradiations were performed in the expected brain regions. This level of precision was reached for nine rats out of ten. A small angle (few degrees) of one rat head in the anteroposterior axis during the irradiation induced an error of less than 1 mm in the irradiation field positioning.

## 5. Discussion

In this study we developed a new method to perform fast imaging of rat heads allowing a 50 µm accuracy alignment for MRT preclinical neurological studies. Using the filtered pink beam produced by the wiggler source at low magnetic field, and thus avoiding the insertion and alignment of the monochromator, limits the number of slits/motor motions required between imaging and therapy modes. Thanks to this time saving, the complete cycle comprising imaging, alignment, transition to therapy mode and actual MRT irradiation took about 3 min. We have irradiated specific brain regions of the GAERS rats (*e.g.* epileptic foci) to validate this alignment procedure. As displayed on the MR images, the bilateral irradiations were performed in the expected brain structures.

Standard synchrotron radiation medical imaging uses highly monochromatic radiation [(Keyrilainen *et al.*, 2008 ▶) flat Si(333) Bragg crystals, energy bandwidth (bw) ×10^−4^], quasi-monochromatic X-ray beams [(Alric *et al.*, 2008 ▶) bent Laue crystals, bw ×10^−3^] or even larger energy spread radiation [(Adam *et al.*, 2003 ▶; Bayat *et al.*, 2009 ▶; Bertrand *et al.*, 2005 ▶) single bent Laue crystal, energy bw ×10^−2^]. The aim of the monochromatic imaging is often to obtain quantitative data in order to measure the concentration of a contrast agent (*e.g.* iodine, xenon or gadolinium) injected in the circulatory system of the animal. MRT preclinical studies recently moved to brain pathologies in which quantitative contrast agent imaging is not required. Non-cancerous brain lesions, *i.e.* epilepsy or Parkinson disease, are treated by conventional radiosurgery and then might be relevant pathologies for MRT applications as well (Sims *et al.*, 1999 ▶; Kurita *et al.*, 2000 ▶; Hadjipanayis *et al.*, 2001 ▶; Romanelli & Anschel, 2006 ▶; Tamura *et al.*, 2009 ▶). For instance, radiosurgical neuro-ablation of the subthalamic nucleus might be a good alternative to the very expensive deep brain stimulation-based treatment in Parkinson’s disease. However, preclinical validation of these assumptions requires a high-precision alignment and targeting procedures which were not available in MRT studies until now. In such diseases, no diffusion and accumulation of the imaging contrast agent are foreseen since normal brain capillaries exhibit a non-permeable blood brain barrier (Cornford & Hyman, 1999 ▶). Thus, the pink-beam imaging method described in this work for non-cancerous brain pathologies presents numerous advantages over commonly used monochromatic imaging. It simplifies considerably the imaging/therapy transitions. Indeed, the insertion of the monochromator into the beam is a complicated and long-lasting operation (∼20 min), as several actuators have to be tuned. Since the monochromator deviates the beam vertically by 15 mm relative to the irradiation white-beam axis, the whole set-up including slits, attenuators and shutters must be lifted to this monochromatic beam position. The two monochromator crystals must be inserted and their orientation carefully tuned in order to obtain a homogeneous monochromatic beam. Some time may also be lost in reaching the necessary thermal stability of the crystals. The main difficulty is then to obtain an exact correlation between the monochromatic beam and the filtered white synchrotron radiation beam used for the treatment in such a way that the image corresponds precisely to the irradiation field. This is particularly the case in preclinical studies where high numbers of specimens are imaged and then irradiated which requires multiple moves between the two modes, hence degrading the positioning accuracy of the brain region target.

The procedure described in this work overcomes all these problems. Indeed, the main advantage of this imaging method is the fact that the beam position is similar and the spectrum is adapted in both therapy and imaging modes. The photon flux reduction is simply obtained by opening the wiggler gap instead of inserting the whole monochromator instrument into the beam path. The gap aperture from 24.8 (1.6 T) to 120 mm (0.13 T) reduced the photon flux by a factor of 6 × 10^3^. The photon flux obtained in a single Laue monochromator used for human angiography was typically 6 × 10^11^ photons s^−1^ mm^−2^, providing a skin dose of 32 mGy delivered for 1 ms. In the present case, the intensity of the pink beam was adjusted to obtain similar detector grey levels dynamics and exposure times, indicating that the dose delivered was of the same order of magnitude as the human synchrotron angiography clinical studies (Bertrand *et al.*, 2005 ▶).

The whole procedure between rat positioning on the goniometer and brain irradiation takes less than 3 min. Moreover, no movement of attenuators and filters are required in this new imaging at open wiggler gap since the same set of attenuators produced satisfactory spectra in both therapy and imaging mode. The quality of the radiographic image obtained with the pink beam is comparable with images obtained with monochromatic beams. Indeed, the pink-beam spectrum extends from 35 to 60 keV, as shown in Fig. 1 ▶. and is centred at about 46 keV which is in an energy range well adapted for small-animal X-ray imaging. The resulting contrast provides an image quality allowing the precise localization of the targeted brain regions according to the skull structures. The principal anatomical reference points, such as bregma and lambda, are clearly identifiable on these X-ray images. Moreover, the properties of the pink beam would not prevent performing contrast agent enhancement imaging, as the *K*-edges of iodine, xenon and gadolinium are 33.17 keV, 34.56 keV and 50.24 keV, respectively, and fall within this spectrum. Therefore, this new imaging modality opens interesting perspectives in the field of image-guided tumor irradiations based on contrast-agent-enhanced tomographic imaging. Such an imaging method does not need to be quantitative; only tumor localization will be required to define the planning of the target volume and tumor margins.

The imaging/alignment procedure described in this work allows highly reproducible and confined three-dimensional irradiations. Indeed, nine GAERS rats out of ten received well targeted irradiations (thalamus or cortex irradiations). The rat head angle alignment in the AP axis was not checked in the last animal which induced an error of a few millimetres in the irradiation site. However, this can be easily corrected by performing a scan at different angles of rotation along the AP axis; the horizontality of the skull will be achieved at the angle where the skull appears the thickest on images (or highest absorption). To our knowledge, our work constitutes the first image-guided microbeam irradiations. Hence, thanks to the association of interlaced MRT (Serduc *et al.*, 2010 ▶) and this alignment procedure, the ID17 biomedical beamline provides a unique tool allowing brain radiosurgery trials on animal patients. The sharp lateral dose fall-off of the synchrotron light is about 180 folds higher than the best current clinical devices such as the gamma knife (Serduc *et al.*, 2010 ▶). Indeed, the function of a given brain region could be studied by selective radiosurgery ablation without impairing surrounding normal tissues. This represents a critical step in MRT preclinical studies and could significantly increase the effectiveness of microbeam irradiations at synchrotron facilities by optimizing the irradiation field size and the positioning, decreasing thereby the neuro-radiotoxicity.

## 6. Conclusion

In this work we described an original method for animal positioning before brain microbeam radiosurgery, using pink-beam imaging. This procedure allows a 50 µm-accuracy alignment with a short transition time between imaging and therapy modalities and *vice versa*. The combination of three-dimensional irradiation and pink-beam imaging alignment appears as a useful and efficient tool in brain pathology radiation therapy. Future developments, *e.g.* tomography and pink-beam contrast agent imaging, will enlarge the potential use of synchrotron-generated X-rays to image and treat cancerous diseases.

## Footnotes

1 Available at http://www.esrf.fr/Accelerators/Groups/InsertionDevices/Software/Xray.

2 All operative procedures related to animal care strictly conformed to the Guidelines of the French Government with licenses 380324 and A3818510002.
